# Dietary Supplementation with Methionine and Lysine Enhances Antioxidant Function and Muscle Quality of Hefang Crucian Carp (*Carassius auratus*)

**DOI:** 10.3390/ani16111636

**Published:** 2026-05-27

**Authors:** Xiao Chen, Yiren Wang, Xubing Wang, Minggui Jiang, Hui Li, Xingyu Huang, Hanyuan Wang, Qianhong Gu, Yonghua Zhou, Yamei Xiao

**Affiliations:** 1 College of Life Sciences, Hunan Normal University, Changsha 410081, Chinagqh@hunnu.edu.cn (Q.G.); 2 Hunan Provincial Key Laboratory of Nutrition and Quality Control of Aquatic Animals, Department of Biological and Chemical Engineering, Changsha University, Changsha 410022, China

**Keywords:** methionine, lysine, muscle quality, antioxidant function, Hefang crucian carp

## Abstract

Methionine (Met) and lysine (Lys), as primary limiting amino acids, play important roles in regulating muscle quality in aquatic animals. This study investigated the effects of dietary Met and Lys supplementation on the growth performance, antioxidant function, and muscle quality of Hefang crucian carp (HCC) using physiological and transcriptomic analyses. Fish were fed three diets for 8 weeks: a basal diet (LA) and two diets supplemented with DL-methionine at 1.7% (MA) and 3.4% (HA), respectively, while L-lysine supplementation was fixed at 3.4%. The results indicated that dietary Met and Lys supplementation had no significant effect on the growth performance of HCC (*p* > 0.05), but significantly reduced serum triglyceride (TG) and cholesterol levels (T-CHO) (*p* < 0.05). Additionally, serum total protein (TP) content was significantly increased in the MA group (*p* < 0.05). Analysis of serum antioxidant enzyme activities indicated that appropriate Met and Lys supplementation improved antioxidant capacity and upregulated the expression of antioxidant-related genes (*Nrf2*, *GPX1a*, *GSTO1*, *GSTP1*) in the muscle. Moreover, the MA group exhibited superior muscle hardness and gumminess, while the HA group had higher springiness and chewiness (*p* < 0.05). Muscle fiber density was significantly increased, whereas diameter and area showed opposite trends in fish fed 1.7% Met and 3.4% Lys (*p* < 0.05). Furthermore, appropriate Met and Lys supplementation significantly affected muscle fiber development genes (*MyoD*, *MyoG*, and *MRF4)*, with *MyoG* highest in the HA group and *MyoD* and *MRF4* highest in the MA group (*p* < 0.05). Comparative transcriptomic analysis of muscle tissue showed DEGs were mainly enriched in pathways correlated with muscle quality, involving cardiac muscle contraction, actin cytoskeleton regulation, PPAR signaling pathway and ECM–receptor interaction. Taken together, our findings enhance the understanding of the molecular mechanisms underlying the effects of dietary Met and Lys on muscle quality in HCC, providing valuable insights for the development of nutritional strategies in the aquaculture industry.

## Simple Summary

Farmed fish rely on high-quality diets to grow well and produce nutritious meat. Methionine and lysine play important roles in regulating muscle quality in aquatic animals. This study aimed to understand how adding these two specific amino acids to the feed of Hefang crucian carp affects their overall health and meat quality over 8 weeks. While the added amino acids did not change growth rates, they significantly improved internal health by reducing blood fat levels and enhancing natural antioxidant defenses. Crucially, the correct balance of methionine and lysine improved the physical texture of the fish filet, making the meat firmer and chewier. These improvements were associated with changes in muscle structure at a microscopic level and biological processes related to muscle development. In conclusion, appropriate supplementation of methionine and lysine can improve fish health and meat quality. This provides useful information for optimizing fish feed formulations and may help produce tastier, higher-quality fish for consumers.

## 1. Introduction

With the rapid development of intensive and large-scale aquaculture models, aquaculture density continues to increase, exerting a profound impact on fish growth, immune function, and especially meat quality—a key criterion for consumers when purchasing aquatic products [1,2]. Meat quality evaluation of aquatic animals mainly includes two aspects: texture and flavor. The quality of muscle is most directly reflected in its texture and muscle fiber characteristics [3]. Among them, texture characteristics directly determine the edibility and palatability of fish, which is the key to consumers’ sensory experience [4]. The core characteristics of muscle fiber, such as diameter and density, are the internal structural basis for the regulation of texture traits such as muscle tenderness, which together constitute the core dimensions of fish quality evaluation [5,6]. Dietary nutritional supplementation can enhance the antioxidant capacity of aquatic animals and improve the tissue structure of muscle fibers, thereby improving muscle quality, with amino acid-based protein synthesis playing a central role [7,8]. Therefore, in the present study, muscle quality is defined as a composite functional and nutritional trait integrating physical texture characteristics, muscle fiber structure, and physiological and biochemical stability. The combined evaluation of these parameters reflects both consumer sensory attributes and the underlying tissue homeostasis under different dietary conditions.

Protein is a core nutrient for fish growth and development, and the digestion and absorption efficiency of protein and the balance of amino acid composition directly affect the growth performance, metabolic level and muscle quality of fish [9]. The widespread use of plant protein sources in recent years often leads to deficiencies in essential amino acids, especially methionine (Met) and lysine (Lys) [10,11]. Consequently, Met and Lys are commonly regarded as the primary limiting amino acids for cyprinids, such as crucian carp, when fed these plant protein-based diets [12]. Their deficiency can ultimately limit fish growth and reduce feed efficiency. Previous studies have shown that Met, as a sulfur-containing amino acid, is not only involved in muscle fiber development and collagen synthesis in fish, but also plays an important role in methylation reactions, antioxidant and lipid metabolism regulation [13,14,15,16]. Moreover, appropriate levels of dietary Lys can promote meat quality and development of fish muscle fibers, and it has irreplaceable functions in promoting lipid metabolism, enhancing immunity and antioxidant capacity, increasing muscle protein deposition, and regulating intestinal digestive enzyme activity [17,18,19,20]. However, existing studies have mostly focused on the effects of single amino acid supplementation, whereas research regarding the combined effects of Met and Lys remains scarce. As the primary limiting amino acids in typical plant-based aquafeeds, the supplementation of one often alters the physiological demand for the other to maintain an optimal amino acid balance. Consequently, the effects of their combined supplementation on the physiological homeostasis and muscle quality of HCC remain largely unclear.

Hefang crucian carp (HCC) is a new national diploid aquatic species bred through multi-step breeding technology [21]. It has the characteristics of fast growth rate, strong resistance and delicious meat, and is widely promoted in the market at present, which is of great significance in the protection of crucian carp germplasm resources [22]. As a cyprinid species, HCC shares similar nutritional characteristics with other crucian carp. Previous studies in cyprinids have shown that dietary Met and Lys are closely associated with growth performance, protein metabolism, and muscle quality, with optimal levels required to maximize growth and feed utilization [23,24]. Notably, under plant protein-based diets, supplementation with limiting amino acids, particularly Met and Lys, can improve growth performance and muscle-related traits in gibel carp (*Carassius auratus gibelio*) [12]. However, such information remains limited in HCC, particularly regarding muscle quality.

Therefore, this paper aims to evaluate the differences in the effects of exogenous amino acid nutritional supplementation on the muscle quality of HCC by the combined supplementation of Met and Lys on the texture characteristics, tissue morphology, and expression of muscle quality-related genes (muscle fiber development and antioxidation) of HCC muscle. The results provide some theoretical basis and practical reference for improving the meat quality and flavor of HCC and optimizing the formula of *Carassius crucian* feed.

## 2. Materials and Methods

### 2.1. Diet Formulation and Experimental Procedure

A commercial feed purchased from Tianbang Feed (Ningbo, Zhejiang, China) was used as the basal diet and served as the control group (LA). Experimental diets were prepared by further supplementing the basal diet with different levels of Met (1.7% for MA and 3.4% for HA), while keeping the additional Lys supplementation constant at 3.4% for both MA and HA groups. The supplemented Met levels were selected based on reported dietary requirement ranges for crucian carp and were set to establish a graded supplementation range, while Lys was maintained at a relatively high level to provide a non-limiting background [25]. All diets were provided as 1.5 mm extruded pellets. All crystalline amino acids (purity ≥ 98%) were sourced from Yuanye Biotech (Shanghai, China). Detailed supplementation levels and proximate compositions are provided in Table 1 and Table 2.

All experimental fish utilized in the feeding trial were sourced from Hunan Normal University Wangcheng Breeding Base (Changsha, Hunan, China). Prior to the formal culture experiment, all fish were acclimated in an indoor fiberglass tank (1500 L) for one week. Subsequently, 135 apparently healthy Hefang crucian carp with an average body weight of (100.0 ± 2.5) g were randomly cultured in 9 fiberglass tanks (90 L), with three tanks serving as one replicate group (15 fish per tank). The experimental conditions were: water temperature (25.5 ± 1.0) °C, dissolved oxygen levels around 6.5 mg/L, ammonia nitrogen concentration below 0.5 mg/kg, pH maintained between 6.5 and 7.0, and a 12 h light–dark cycle (light from 8:00 to 20:00). Fish were fed two times daily at 8:00 and 16:00 until reaching obvious satiety, defined as cessation of active feeding and fish ignoring the offered feed (typically within 5–10 min). Throughout the experiment, one-fifth of the water was replaced daily to maintain water quality. The aquaculture was conducted using an indoor recirculating water system for an eight-week period.

### 2.2. Sample Collection

After 24 h of fasting, all experimental fish were anesthetized via immersion in MS-222 (60 mg/L; E10521, Sigma-Aldrich, St. Louis, MO, USA) prior to dissection. Subsequently, six fish per group (two fish from each replicate tank) were randomly collected for blood and tissue sampling. Blood samples were drawn from the caudal vein and kept at 4 °C overnight, followed by centrifugation at 4500 rpm for 10 min. The supernatant (serum) was then collected and stored at −80 °C. Following dissection with sterilized scissors, viscera and liver tissues were excised and weighed to calculate organ indices. Tissue samples were immediately frozen in liquid nitrogen and then stored at −80 °C until further analysis. All procedures were performed on ice. Muscle samples (2 cm × 2 cm × 1 cm) were fixed in 4% paraformaldehyde at room temperature for the subsequent detection of muscle fiber characteristics. In addition, 1 cm × 1 cm × 1 cm dorsal muscle samples were taken from both sides of the back of the same fish for muscle texture analysis and tested immediately on the same day. Consequently, a total of six independent biological replicates (*n* = 6) per group were utilized for subsequent analyses, including serum biochemical parameters, antioxidant enzyme assays, qPCR, and texture analysis.

### 2.3. Analysis of Serum Biochemical Parameters

The serum biochemical indices, including total protein (TP), glucose (GLU), triglycerides (TG) and cholesterol (T-CHO) were measured using the matching commercial reagent kits (A045-4, A145-1-1, A110-1-1, A111-1-1; Nanjing Jiancheng Bioengineering Institute, Nanjing, China). Serum malondialdehyde (MDA), total antioxidant capacity (T-AOC) and superoxide dismutase (SOD) were analyzed using their corresponding kits (A003-1-2, A015-2-1, A001-3; Nanjing Jiancheng Bioengineering Institute, Nanjing, China). All experiments were performed strictly following the manufacturer’s instructions.

### 2.4. Texture Analysis of Muscle

Two fish were randomly selected from each cage. Back muscle samples (1 cm^3^) of uniform thickness and size were excised from the same anatomical location on both sides of each fish. Texture measurements from the left and right sides were averaged to obtain a single value per fish, which was used as one biological replicate for statistical analysis (*n* = 6 per group). Texture profile analysis (TPA) was immediately performed to determine the hardness, cohesiveness, springiness, gumminess, chewiness and adhesiveness of the muscle samples (TMS-PRO, Food Technology Corporation, Sterling, VA, USA). The TPA parameters were set as follows: initial force 0.1–250 N, probe height 1 cm, descending speed 30 mm/min, and deformation 60%. Each sample’s textural characteristics were calculated via the force–time curve produced using the computer software, Texture Lab Pro (1.18-408, FTC, Sterling, VA, USA).

### 2.5. Histological Observation

The fixed muscle samples were embedded in paraffin and stained with hematoxylin and eosin, then washed with 70% alcohol. After HE staining and mounting, muscle tissue sections were scanned using a PANNORAMIC MIDI digital slide scanner (3DHISTECH Ltd., Budapest, Hungary). The ImageJ software (v1.54p, NIH, Bethesda, MD, USA) was used to identify muscle fiber characteristics. For each sample section, 20 myofibers were randomly selected to quantify the myofiber diameter and three non-overlapping fields of view were counted for myofiber number. Myofiber density (fibers/mm^2^) was calculated as the total number of counted myofibers divided by the area of the counted field.

### 2.6. Transcriptome Analysis of Muscle

After RNA extraction and purification, libraries were constructed from three biological replicates per group (*n* = 3) and sequenced by Shanghai Bioprofile Technology Company Ltd. (Shanghai, China) using the Illumina HiSeq 2500 platform. RNA-seq quality metrics and mapping statistics for each sample are provided in Supplementary Tables S1 and S2. To identify differentially expressed genes (DEGs) among the experimental groups, the expression level of each transcript was quantified using the fragments per kilobase of transcript per million mapped reads (FPKM) method. *p*-values were adjusted using the false discovery rate (FDR) method (Benjamini–Hochberg correction) to control for false positives. Consequently, the criteria for identifying DEGs were defined as genes with an FDR < 0.05 and |log_2_FoldChange| ≥ 2. Gene function annotation was based on the following databases: GO (Gene Ontology), KEGG (Kyoto Encyclopedia of Genes and Genomes), NR (NCBI non-redundant protein sequences), KOG/COG (Clusters of Orthologous Groups of Proteins), Pfam (Protein family) and Swiss-Prot (manually annotated and reviewed protein sequence database). The raw transcriptome sequencing data generated in this study have been deposited in the NCBI Gene Expression Omnibus (GEO) database under the accession number GSE331020.

### 2.7. Quantitative Real-Time PCR

Gene-specific qPCR primers were designed with Primer 5.0 software (Table 3), and *β-actin* served as the internal control for data normalization. The stability of *β-actin* was verified prior to normalization, with Ct values showing minimal variation among treatment groups. Additionally, the amplification performance and specificity of all primers were systematically evaluated and optimized. Melting curve analysis confirmed a single, sharp, specific peak for each amplicon, demonstrating the absence of non-specific products and primer-dimer formation. Total RNA was extracted by homogenizing muscle tissue in 1 mL Trizol (Takara, Kusatsu, Japan) using a low-temperature high-speed homogenizer (Thermo Fisher Scientific, Waltham, MA, USA). RNA quality and integrity were assessed via 1.5% agarose gel electrophoresis and a full-wavelength microplate reader (Thermo Fisher Scientific) with DEPC water as the blank control. First-strand cDNA was synthesized from 1 μg of qualified RNA using the PrimeScript II 1st Strand cDNA Synthesis Kit (TaKaRa, Kusatsu, Japan) according to the manufacturer’s instructions.

Each qPCR reaction was prepared in a total volume of 10.0 μL, consisting of 1.0 μL of diluted cDNA (1:10), 0.2 μL each of 10 μM forward and reverse primers, 5.0 μL of SYBR Green Pro Taq HS Premix (Cat. No. AG11718; Accurate Biology, Changsha, China), and ddH_2_O was subsequently added to bring the total volume to 10.0 μL. The PCR conditions included initial denaturation at 95 °C for 30 s, followed by 40 cycles of denaturation at 95 °C for 3 s, annealing at 60 °C for 25 s, and extension at 72 °C for 10 s. All assays were performed on a Bio-Rad CFX96™ Real-Time PCR System (Bio-Rad, Hercules, CA, USA). Relative expression levels were quantified using the comparative Ct (2^−ΔΔCt^) method with six biological replicates per group, each analyzed in three technical replicates.

### 2.8. Statistical Analysis

All data are expressed as mean ± SEM. Prior to parametric statistical analysis, the Shapiro−Wilk test was employed to verify the normality of data distribution, and Levene’s test was used to evaluate the homogeneity of variances. One-way analysis of variance (ANOVA) followed by Duncan’s multiple comparison test was used to assess differences in means using the SPSS 27.0 software (Chicago, IL, USA). All experiments were performed with at least three biological replicates. A *p* value < 0.05 was set as the threshold for statistical significance. A post hoc power analysis was conducted using G·Power software (version 3.1.9.7) to evaluate the statistical power of the growth performance analysis.

## 3. Results

### 3.1. Growth Performance and Somatic Indices of HCC

To analyze the effects of Met and Lys supplementation on the growth of HCC, growth performance-related indicators were measured and statistically analyzed (Table 4). The results showed that with the gradual increase in amino acid concentrations, the weight gain rate and specific growth rate of HCC tended to increase, but no statistically significant differences were observed. Survival rate remained unaffected across all groups. Additionally, no significant changes were detected in the condition factor, viscerosomatic index, or hepatosomatic index of HCC among different treatment groups. Although the dietary treatments did not induce significant improvements in growth performance, subsequent physiological and histological evaluations were conducted to determine if these amino acid supplementations altered internal metabolic and structural parameters.

### 3.2. Serum Biochemical Analysis

As shown in Table 5, dietary supplementation with moderate levels of Met and Lys significantly elevated the total protein (TP) content in HCC (*p* < 0.05), whereas the glucose (GLU) content exhibited a non-significant upward trend. In contrast, a further increase in Met and Lys concentrations resulted in a marked increase in GLU content. Additionally, compared with the non-supplemented control group, serum total cholesterol (T-CHO) and triglyceride (TG) contents in HCC were significantly reduced in all amino acid-supplemented groups (*p* < 0.05).

### 3.3. Serum and Muscle Antioxidant Capacity Analysis

**Figure 1:**
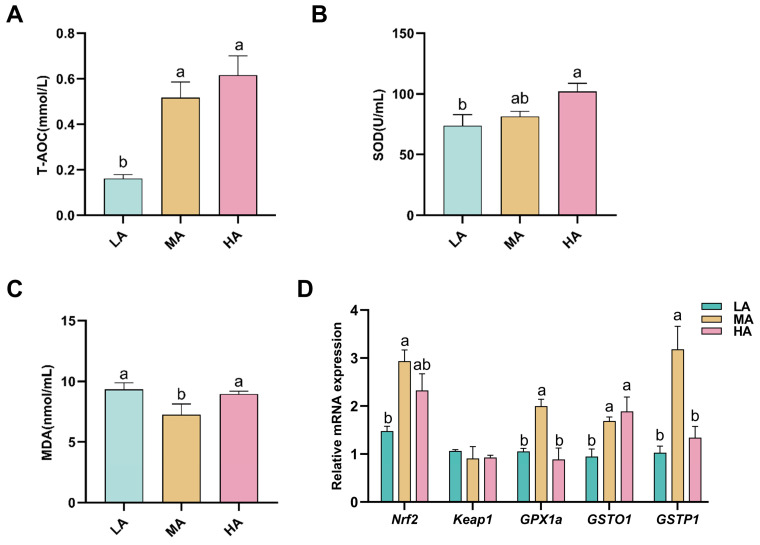
Serum and muscle antioxidant capacity of Hefang crucian carp fed different diets for 8 weeks. (**A**) total antioxidant capacity (T-AOC); (**B**) superoxide dismutase (SOD); (**C**) malondialdehyde (MDA); (**D**) Relative expressions of genes involved in muscle antioxidant in the muscle of Hefang crucian carp. The results are shown as mean ± SEM (*n* = 6); Different letters indicate significant differences (*p* < 0.05). The exact *p*-values obtained from the overall one-way ANOVA were as follows: (**A**) *p* < 0.001, (**B**) *p* = 0.035, (**C**) *p* = 0.034; and for genes in (**D**): *Nrf2* (*p* = 0.017), *Keap1* (*p* = 0.209), *GPX1a* (*p* = 0.002), *GSTO1* (*p* = 0.036), and *GSTP1* (*p* = 0.006).

As shown in Figure 1A,B, the activities of antioxidant enzymes, including total antioxidant capacity (T-AOC) and superoxide dismutase (SOD), were significantly elevated with increasing amino acid concentrations. In contrast, the malondialdehyde (MDA) content exhibited a reverse trend, with its lowest level observed in the MA group (Figure 1C). We further detected the mRNA expression profiles of antioxidant-related genes in muscle tissues. As illustrated in Figure 1D, the expression levels of nuclear factor erythroid 2-related factor 2 (*Nrf2*), glutathione peroxidase 1a (*GPX1a*), and glutathione s-transferase pi 1 (*GSTP1*) were significantly upregulated following supplementation with moderate concentrations of Met and Lys (*p* < 0.05), whereas their expression declined with further increases in amino acid supplementation levels. Notably, glutathione s-transferase omega 1 (*GSTO1*) expression was significantly induced in both the Met and Lys treatment groups (*p* < 0.05). By contrast, no statistically significant difference was observed in Kelch-like ECH-associated protein 1 (*Keap1*) expression across all experimental groups, whereas all exhibited a tendency to decrease. 

### 3.4. Muscle Instrumental Texture Analysis

The muscle texture analysis of HCC fed the experimental diets is presented in Table 6. The results indicated that dietary supplementation with different concentrations of Met and Lys induced significant alterations in muscle hardness, springiness, gumminess, and chewiness (*p* < 0.05), whereas no marked difference was observed in muscle cohesiveness and adhesiveness across all treatment groups. Specifically, the MA group exhibited superior muscle hardness and gumminess, while the HA group had higher springiness and chewiness.

### 3.5. Morphology of Myofibers

**Figure 2:**
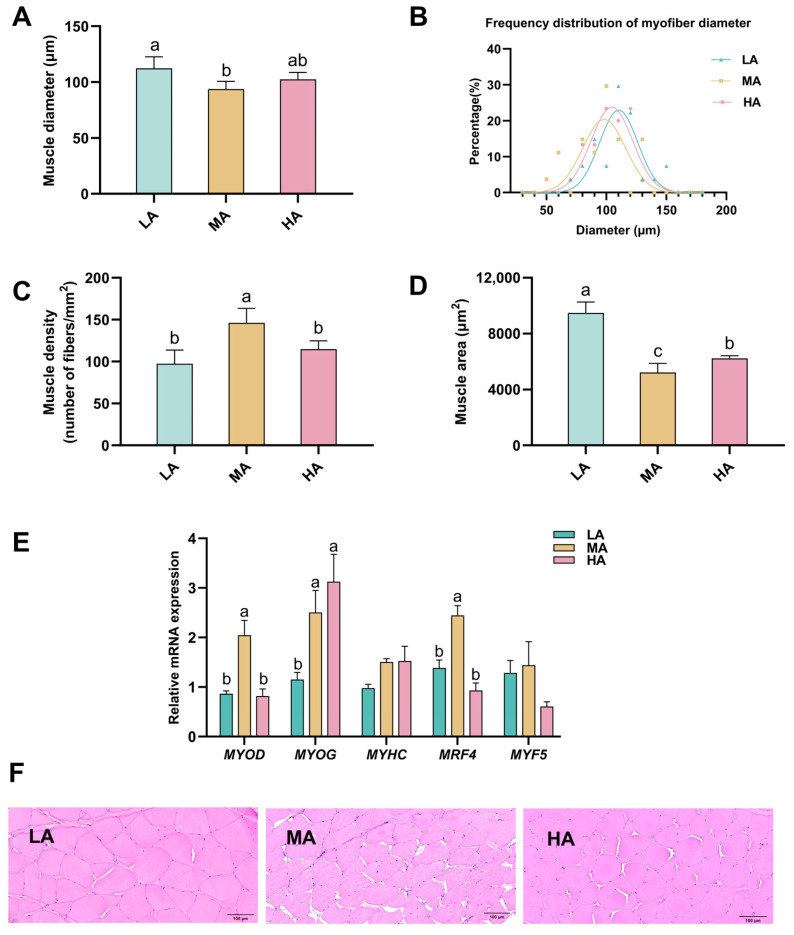
The effects of dietary Met and Lys supplementation on the development of muscle fiber characteristics in Hefang crucian carp. (**A**–**D**) Myofiber morphological indices and frequency distribution of myofiber diameter. (**E**) Relative expressions of genes involved in muscle fiber development in the muscle of Hefang crucian carp. (**F**) Histological morphology of myofibers (scale bar = 100 μm). The results are shown as mean ± SEM (*n* = 6). Different letters indicate significant differences (*p* < 0.05). The exact *p*-values from the overall one-way ANOVA are as follows: (**A**) *p* = 0.03, (**C**) *p* = 0.02, (**D**) *p* < 0.001; and for genes in (**E**): *MYOD* (*p* = 0.007), *MYOG* (*p* = 0.023), *MYHC* (*p* = 0.13), *MRF4* (*p* < 0.001), and *MYF5* (*p* = 0.217).

As shown in Figure 2A,D, the myofiber diameter and cross-sectional area in all treatment groups were significantly smaller than those in the control group. In contrast, muscle fiber density exhibited an opposing trend, with the maximum density observed in the MA group (Figure 2C). Furthermore, Figure 2B reveals that larger myofibers (>115 μm) predominate the LA group, whereas smaller myofibers (<100 μm) were more abundant in the treatment groups. Cross-sectional observations of skeletal muscle fibers across all groups demonstrated an irregular polygonal morphology, with no significant intergroup differences in shape (Figure 2F). The white interstitial regions correspond to the endomysium, a loose connective tissue layer that individually encapsulates each muscle fiber [26]. In addition, myogenic regulatory factors (MRFs) were measured, the mRNA expression of Myogenin (*MyoG*) in the muscle of HCC was significantly upregulated with increasing amino acid concentrations (*p* < 0.05), as illustrated in Figure 2E. Notably, Myogenic differentiation 1 (*MyoD*) and myogenic regulatory factor 4 (*MRF4*) exhibited similar trends, with their expression levels peaking in the MA group. There were no significant differences in myosin heavy chain (*Myhc*) and myogenic factor 5 (*Myf5*) gene expression among groups (*p* > 0.05). However, compared to the LA group, there was a trend of increasing *Myhc* and *Myf5* gene expression when the Met and Lys addition amount was 1.7% and 3.4%.

### 3.6. Transcriptomic Analysis of Flesh by Dietary Methionine and Lysine Levels

**Figure 3:**
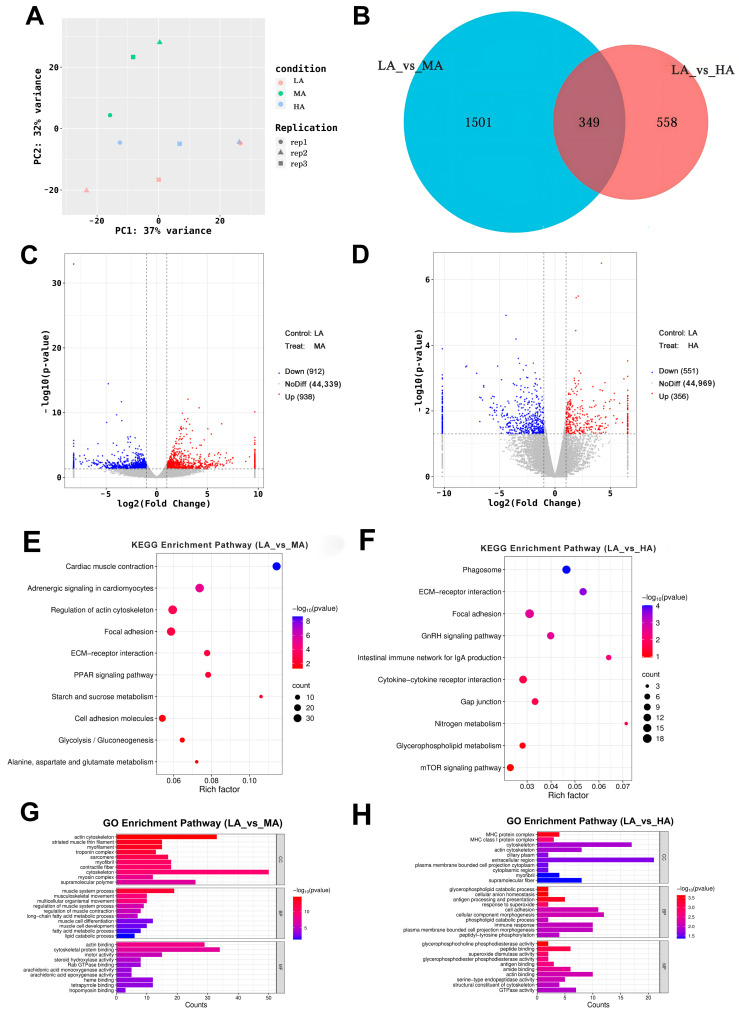
Transcriptome analysis of muscle from Hefang crucian carp fed different diets for 8 weeks (*n* = 3). (**A**) PCA plot of muscle transcriptomes. (**B**) Venn diagram of DEGs. (**C**,**D**) Volcano plots showing DEGs in muscle between different comparison groups. The gray dots represent the DEGs that are not differentially expressed, the red dots represent the upregulated DEGs, and the blue dots represent the downregulated DEGs. (**E**,**F**) Top 10 KEGG enrichment pathways in the different comparison groups. (**G**,**H**) Top 10 most significantly enriched GO terms in the MF, BP, and CC categories across different comparison groups.

To further investigate the regulatory effects of dietary Met and Lys on the muscle gene network of HCC, transcriptome analysis was performed on the three experimental groups (Figure 3). Principal component analysis (PCA) revealed significant differences in gene expression profiles among the three groups, verifying the feasibility of subsequent analyses (Figure 3A). Compared with the LA group, the MA group exhibited distinct expression patterns, with 938 genes significantly upregulated and 912 genes significantly downregulated (Figure 3C). In addition, 551 genes were downregulated and 356 genes were upregulated in the HA group relative to the LA group (Figure 3D). Among these differentially expressed genes (DEGs), 349 genes were identified as common DEGs across pairwise comparisons (Figure 3B). The analysis of KEGG enrichment indicated that pathways associated with muscle quality and flavor including cardiac muscle contraction, regulation of actin cytoskeleton, and PPAR signaling pathway were enriched in the MA group (Figure 3F). Additionally, enrichment of ECM–receptor interaction and focal adhesion pathways suggested altered structural stability of muscle tissue, a factor closely linked to muscle water-holding capacity and textural properties [27]. For the LA vs. HA comparison (Figure 3F), glycerophospholipid metabolism and the mTOR signaling pathway were enriched. Consistent with the LA vs. MA comparison, ECM–receptor interaction pathway was again enriched, reinforcing that dietary Met and Lys supplementation modulates the structural integrity of muscle tissue. For the GO enrichment analysis results of DEGs, we categorized the terms into molecular function (MF), biological process (BP), and cellular component (CC). The top 10 most significantly enriched GO terms (with the smallest *p*-values) in each category were selected for display, and the results are presented in Figure 3G,H. Compared to the LA group (Figure 3G), the most significantly enriched BP terms included muscle contraction, actin filament organization, and cell adhesion. At the MF level, DEGs were most related to “binding” and “catalytic activity”. In the comparison between LA and HA (Figure 3H), key enriched BP terms involved lipid metabolic processes and cell–matrix adhesion. Meanwhile, MF terms such as phospholipid binding and protein kinase activity were significantly enriched.

### 3.7. Gene Expression Verified by Quantitative Real-Time PCR

**Figure 4:**
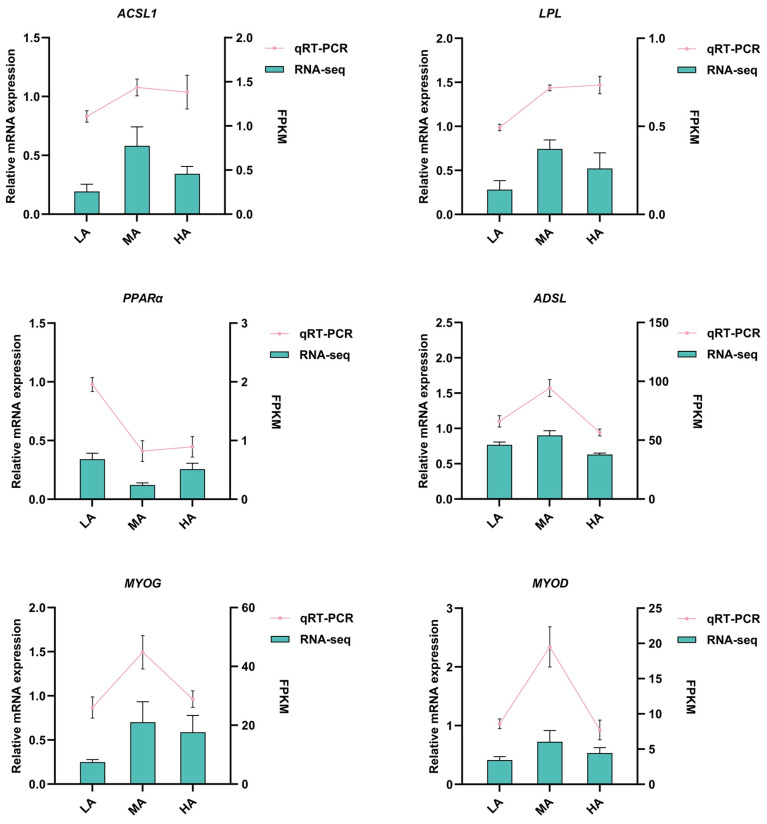
qPCR validation of DEGs related to muscle lipid deposition and fiber development identified from KEGG enrichment analysis. ACSL1: Acyl-CoA synthetase long-chain family member 1; LPL: Lipoprotein lipase. PPARα: Peroxisome proliferator-activated receptor alpha; ADSL: Adenylosuccinate lyase; MyoD: Myogenic differentiation 1; MyoG: Myogenin.

To verify the reliability and accuracy of the RNA-seq data, we performed qRT-PCR analysis on genes related to muscle development and lipid metabolism. The expression trends observed in the qRT-PCR analysis were consistent with those of the RNA-seq results (Figure 4), further confirming the reliability of the transcriptome data.

## 4. Discussion

### 4.1. Growth Performance Responses and Interspecific Differences

This study investigated the effects of combined supplementation with different proportions of Met and Lys on the growth performance and related body indices of HCC. The results showed that although the weight gain rate and specific growth rate of HCC both exhibited an upward trend with the gradual increase in amino acid supplementation, these differences did not reach statistical significance. This may be attributed to the possibility that the basal diet already met the basic nutritional requirements of the fish. Therefore, the observed changes in physiological and muscle quality-related parameters are more likely to reflect physiological modulation rather than correction of a nutritional deficiency [28]. In addition, a post hoc power analysis indicated low statistical power (0.43), suggesting that relatively high variability among individuals and the limited sample size may have been insufficient to detect significant differences despite the observed increases [29,30]. A similar phenomenon of non-significant growth enhancement has also been reported in gibel carp (*Carassius auratus gibelio*) when supplemented with either crystalline or coated forms of Met and Lys [31]. Likewise, studies have shown that additional dietary supplementation of Met and Lys does not provide extra benefits for broiler production performance [32]. In contrast to our findings, a study on grass carp (*Ctenopharyngodon idella*) demonstrated a significant increase in growth performance and protein efficiency ratio (PER) in response to elevated dietary Met, which subsequently decreased beyond an optimal level [33]. These discrepancies may be attributed to differences in species, dietary composition, amino acid levels, experimental duration and conditions. Furthermore, the relatively large initial body size of the fish used in the present study may reduce the sensitivity for detecting subtle growth responses to dietary amino acid manipulation.

Taken together, the present results suggest that dietary Met and Lys supplementation showed a tendency to improve growth performance under the current experimental conditions. However, further studies with larger sample sizes are required to confirm this effect. Importantly, notable changes were observed in physiological and muscle quality-related parameters, suggesting that these amino acids may primarily influence metabolic and cellular processes prior to measurable changes in growth. Furthermore, from a practical perspective, this targeted improvement in muscle quality aligns with the industry’s shift towards producing value-added, premium aquatic products.

### 4.2. Alterations in Serum Biochemical Parameters and Physiological Significance

Blood biochemical indexes serve as crucial indicators for evaluating the physiological and metabolic functions, nutritional status, and overall health of fish [34]. Among them, serum total protein (TP) is a key indicator for assessing the metabolic state of body protein and an increase in its content facilitates enhanced protein deposition in the organism [26]. Notably, alterations in this index are closely associated with environmental adaptation, amino acid metabolism, and other regulatory factors [35]. In this experiment, the TP content in serum increased significantly after Met and Lys supplementation, suggesting enhanced protein deposition and potentially supporting tissue growth and development. Similar trends have been reported in *Plectropomus leopardus* and *juvenile gibel carp* [17,36]. Although these studies involve different species, crucian carp may exhibit comparable TP response patterns due to the conserved nature of amino acid-driven protein synthesis in teleosts, particularly among closely related cyprinids such as gibel carp. Nevertheless, species-specific differences in amino acid metabolism may still influence the magnitude of these TP responses.

As an important indicator of nutritional status in fish, serum glucose (GLU) level typically indicates that fish are actively feeding and in good health when it increases within a reasonable range [37]. Supplementation with a moderate ratio of Met and Lys exerted no significant effect on GLU content in HCC. A similar trend has been reported in large Nile tilapia, where concurrent supplementation of Met and taurine showed no obvious effect on serum GLU levels [38], although this comparison is indirect due to differences in amino acid combinations. However, the GLU levels in the HA group was significantly increased when the supplemental level of Met was further increased. This phenomenon may be related to differences in amino acid utilization under varying supplementation levels. At moderate levels, Met and Lys may primarily support growth, development, and muscle protein synthesis, with absorbed amino acids preferentially allocated to protein deposition without excessive accumulation, thereby maintaining glucose metabolic homeostasis [39,40]. In contrast, under excessive amino acid supply, alterations in metabolic pathways may occur. Excess Met and Lys may be involved in amino acid catabolism and could potentially act as gluconeogenic precursors, contributing to the formation of metabolic intermediates such as pyruvate and α-ketoglutarate, which are associated with glucose metabolism [41]. However, the involvement of gluconeogenesis in this process requires further verification, as key gluconeogenic enzymes and insulin signaling pathways were not measured in the present study.

Previous studies have shown that supplementation of certain levels of Met and Lys in aquatic diets can reduce serum levels of TG and T-CHO [38,42,43]. The results of this experiment also indicated that the addition of Met and Lys in the feed could significantly reduce the contents of serum TG and T-CHO in HCC. As essential amino acids, dietary supplementation of Met and Lys may help alleviate amino acid limitations and support muscle protein synthesis, which could be associated with reduced reliance on lipid storage and alterations in lipid metabolism [8]. In contrast to the findings of the present study, a recent study reported that the levels of TG and T-CHO in farmed tilapia juveniles did not change noticeably at different dietary Met levels [44]. Similar results have also been reported in studies on other species [45,46]. This may be due to species differences and the addition of single amino acids in the diet.

### 4.3. Enhancement of Antioxidant Capacity and Regulatory Pathways

Oxidative stress causes meat quality deterioration in fish [47]. This study demonstrated that Met and Lys diets could increase serum SOD and T-AOC, thereby enhancing the antioxidant capacity of HCC. Similar findings were reported in yellow catfish (*Pelteobagrus fulvidraco*) and gibel carp (*Carassius auratus gibelio*) [48,49]. MDA, as a lipid oxidation product, directly reflects physiological oxidative stress [50], which can be induced by dietary Met deficiency in fish [51]. The supplementation of Met and Lys at a moderate level (MA group) significantly reduced serum MDA content, suggesting a potential inhibitory effect on lipid peroxidation and oxidative damage in HCC. However, MDA levels increased again in the HA group, approximating those of the LA group. This dose-dependent response suggests that while moderate supplementation may enhance antioxidant capacity, excessive Met and Lys intake could attenuate these benefits. The rebound in MDA at the higher dose may be associated with metabolic burden induced by excessive amino acid supplementation. Previous studies have indicated that excessive dietary Met can lead to the accumulation of pro-oxidative metabolites, which may promote the generation of reactive oxygen species (ROS) and lipid peroxidation [52]. Therefore, maintaining a moderate supplementation level may be important for optimal redox balance in HCC.

Under physiological homeostasis, *Keap1* binds to *Nrf2* in the cytoplasm and mediates the ubiquitination modification and degradation of the latter [53]. Under oxidative stress, this interaction is disrupted, allowing *Nrf2* to translocate to the nucleus and activate the expression of antioxidant genes, such as *GPX* and *GSTs* [54,55]. Therefore, we further examined the expression levels of genes related to antioxidant capacity in muscle and found that MA group significantly increased the expression of *Nrf2* and its downstream *GPX1a*, *GSTO1* and *GSTP1*. Based on the observation of antioxidant indicators in serum, it was found that the antioxidant capacity of MA muscle was significantly increased, which may be related to the increased expression of *GPX1a*, *GSTP1* and *GSTO1* mRNA activated by Keap1-Nrf2 signaling pathway [56]. Conversely, these expressions were downregulated in the HA group. This attenuation may be attributed to the oxidative stress and metabolic burden induced by excessive dietary Met, which impairs cellular antioxidant defense mechanisms. Consistent with our results, a previous study reported that an optimal Met level (0.84%) activated the Nrf2 pathway in juvenile blunt snout bream (*Megalobrama amblycephala*), whereas excessive Met supplementation (1.28%) failed to yield further antioxidant improvements [57].

### 4.4. Improvement of Muscle Textural Properties and Myogenic Regulatory Mechanisms

Texture characteristics are crucial indicators of the edible quality of flesh products and provide direct measures of muscle palatability. These parameters include springiness, hardness, chewiness, cohesiveness, and gumminess [3]. Dietary supplementation with Met and Lys in the present experiment induced significant changes in muscle texture parameters. Muscle hardness and gumminess were markedly increased in the MA group, while springiness and chewiness were higher in the HA group, indicating differential effects of supplementation levels on distinct texture attributes. These differences may be linked to changes in muscle fiber characteristics, potentially mediated by the regulation of myogenic regulatory factors (MRFs). Additionally, flesh texture is also associated with collagen metabolism. However, specific collagen-related indices were not assessed in the present study. This limitation warrants further investigation to fully elucidate the underlying structural mechanisms.

Previous studies have shown that dietary supplementation with Met alone can reduce the muscle fiber diameter of rice field eel (*Monopterus albus*) and enhance its muscle hardness, gumminess, and chewiness [14], with similar results also reported in grass carp (*Ctenopharyngodon idella*) [13]. Similarly, Lys supplementation alone has been shown to affect muscle fiber characteristics in fish [58]. However, the present study further explored the effects of combined Met and Lys supplementation on muscle texture and fiber characteristics. In the present study, combined supplementation with Met and Lys resulted in decreased muscle fiber diameter and increased fiber density compared with the LA group, which is generally associated with changes in muscle texture [5,59]. However, the effects of MA and HA were not consistent across all texture parameters, suggesting that different aspects of muscle texture may respond differently to varying supplementation levels, and a clear dose–response relationship was not established in the present study. In addition, correlation analyses were not conducted between texture parameters and collagen content or intramuscular fat, which warrants further investigation.

With the increase in amino acid concentration, the mRNA expression of *MyoG* was significantly up-regulated, and the expression of *MyoD* and *Myf5* reached the peak in the MA group, indicating that dietary Met and Lys supplementation could promote myogenesis and myofiber proliferation. As key regulators of muscle development, *MyoD* and *Myf5* are crucial for the initiation of myoblast proliferation and differentiation, while *MyoG* plays a core role in mediating the fusion of myoblasts into mature muscle fibers [60,61]. The elevated expression levels of these MRFs were accompanied by the formation of a greater number of small-diameter muscle fibers, which may be associated with increased muscle fiber density and improved muscle texture. When the amino acid ratio exceeded a certain threshold, the increase in texture parameters and MRFs expression tended to plateau. This observation is consistent with previous findings in juvenile turbot (*Scophthalmus maximus*), where excessive dietary Met did not further enhance MRFs expression and showed no additional effects [62]. This phenomenon may reflect the saturation effect of amino acid-mediated metabolic regulation, whereby additional supplementation cannot produce more significant physiological effects once amino acid supply meets the maximum physiological demand of the body [32,57].

### 4.5. Key Metabolic Pathways Revealed by DEGs

Transcriptome sequencing (RNA-seq) enables the comprehensive analysis of all transcripts in cells under specific physiological conditions, offering novel approaches to identifying key genes and their intricate regulatory mechanisms underlying muscle quality improvement [63]. Compared with LA group, MA group showed the most significant gene expression difference, with a total of 1850 DEGs identified, while the number of DEGs in the HA group was relatively small. These findings suggest a distinct dose-dependent effect of Met and Lys levels on muscle gene expression, indicating that a moderate amino acid supplementation level may be more conducive to inducing the remodeling of genes associated with muscle metabolism.

In KEGG enrichment analysis, pathways such as cardiac muscle contraction, regulation of actin cytoskeleton, and PPAR signaling pathway, all associated with muscle quality, were enriched in MA group. Although the classical function of cardiac muscle contraction is to maintain cardiac contraction homeostasis, its core molecules (actin, myosin, troponin and calcium signaling related genes) are highly conserved in cardiac muscle and skeletal muscle [64].These molecules may influence muscle texture traits, including tenderness and chewiness, possibly through effects on sarcomere organization and muscle fiber characteristics [65]. In addition, Ca^2+^ signaling is known to play a role in muscle development and metabolic regulation, and may be involved in the responses observed in the present study. Previous studies have shown that elevated intracellular Ca^2+^ concentration activates the Calcineurin–NFAT pathway, which regulates the formation of oxidative muscle fibers and ultimately improves muscle quality [66].

The regulation of the actin cytoskeleton pathway is essentially achieved through a dynamic molecular network composed of actin polymers and their associated actin-binding proteins [67]. As demonstrated by previous research in grass carp, pigs and chickens, this signaling pathway is closely associated with muscle growth and development as well as intramuscular fat deposition, both of which are linked to meat quality and flavor [68,69,70]. Met and Lys are regarded as important nutritional regulators of lipid metabolism [71,72].

The present study found that after combined supplementation with Met and Lys, the PPAR signaling pathway, which is closely associated with lipid metabolism, was enriched in muscle tissues. This finding suggested that Met and Lys may be involved in the regulation of lipid metabolic processes in muscle. This interpretation was further supported by the observed decreasing trends in serum TG and T-CHO, indicating systemic alterations in lipid metabolism. However, direct evidence linking these transcriptomic and physiological changes to improvements in muscle flavor is lacking, as flavor-related metabolites (such as free amino acids and volatile compounds) were not measured in the present study.

Additionally, cell conjunction-related pathways, namely ECM–receptor interaction, were significantly enriched in both comparison groups, indicating that the addition of Met and Lys was crucial for maintaining muscle tissue integrity. In this pathway, ECM components can specifically interact with muscle satellite cells to affect cell localization, activation, apoptosis, proliferation and differentiation, and play a key role in the formation of muscle microenvironment [73]. In addition, ECM components can promote the proliferation and differentiation of muscle satellite cells and affect the diameter and density of muscle fibers [74], which is mutually confirmed with our histological observation that Met and Lys diet groups have higher muscle fiber density, smaller diameter and area.

The process of muscle development involves continuous proliferation of new muscle fibers and hypertrophy of muscle fibers, which is executed under the coordinated regulation of multiple biological processes and components [75]. In GO enrichment, muscle contraction, actin filament assembly and cell adhesion were significantly enriched in the MA group compared with LA group, which was mutually confirmed with the KEGG pathway analysis results, highlighting the key regulation of amino acids in the MA group on muscle contraction function and cell structure. Moreover, the enrichment of terms related to “binding” and “catalytic activity” suggested that these DEGs might mediate intermolecular interactions or enzymatic reactions to exert precise regulation over muscle physiological processes. This observation was similar to the previously reported mechanism in pigs, in which mitochondrial respiratory activity and metabolic enzymes were demonstrated to modulate muscle oxidative capacity and fiber-type composition [76]. In the comparison between the LA and HA group, the enrichment of terms including lipid metabolic process, phospholipid binding, cell–matrix adhesion, and protein kinase activity indicated that high-dose amino acid supplementation prioritized the regulation of muscle lipid metabolism and signal transduction processes. These transcriptomic changes are consistent with the observed physiological responses and muscle texture variations, suggesting a coordinated regulation of molecular pathways underlying both biochemical status and flesh quality under different supplementation levels.

Finally, the present study did not employ a factorial design to independently evaluate the effects of methionine (Met) and lysine (Lys). The simultaneous variation in both amino acids between the LA and MA groups, followed by variation in Met alone between the MA and HA groups, limits the ability to distinguish their individual contributions or potential interactions. Therefore, the results should be interpreted as reflecting the combined effects of Met and Lys supplementation. Further studies using factorial or dose–response designs are required to clarify their independent and interactive roles.

## 5. Conclusions

In conclusion, dietary supplementation with Met and Lys modulated antioxidant capacity and muscle quality-related parameters in HCC. Moderate supplementation was associated with improved antioxidant status and favorable changes in muscle texture characteristics. These effects were accompanied by alterations in serum biochemical indices, muscle fiber traits, and the expression of muscle-related genes. Transcriptomic analysis further suggested that these responses may be associated with the regulation of pathways involved in muscle development and lipid metabolism. However, no significant improvement in growth performance was observed under the present experimental conditions, and the individual contributions or interactive roles of Met and Lys could not be distinguished. Overall, this study provides preliminary insights into the potential roles of dietary amino acid supplementation in modulating muscle quality, highlighting its promising application in the production of value-added aquatic products. Further studies are required to validate the underlying mechanisms and the roles of key differentially expressed genes.

## Figures and Tables

**Table 1:** Supplementation levels of amino acids (% of basal diet).

Component		LA	MA	HA
Supplementation level (%) ^1^	DL-Met	0	1.7	3.4
	L-Lys·HCl	0	3.4	3.4

^1^ The values represent the levels of crystalline amino acids supplemented by the researchers into the commercial basal diet. They do not include the baseline levels of methionine and lysine present in the protein ingredients, which were not specified by the manufacturer.

**Table 2:** Nutritional composition of basal diet (% dry matter).

Component	Content (%)
Crude Protein	36.0
Crude Lipid	3.0
Crude Fiber	8.0
Crude Ash	16.0
Total Phosphorus	0.5
Moisture	12.0

**Table 3:** The sequences of the designed primers used in this study.

Genes	Forward Primer (5′-3′)	Reverse Primer (5′-3′)	Accession No.
*Nrf2*	CGGGAGCAGGAGAAAGCA	TGGGCACTGGTCACGGT	XM_026272637.1
*Keap1*	TGGCTCATAAAGTGGTGCTG	TGCCGATGGCGTTGCT	XM_026245355.1
*GPX1a*	GTGGTGCTTATTGAAAATGTGG	TGATGTCCGAACTGGTTGC	XM_074559940.1
*GSTO1*	CAATGCCAAGGGAATCAAA	AAACGGGTCAGACGGGAG	XM_026223937.1
*GSTP1*	CATTTGCGGACTACAACCTG	GGCTTTGATTTTGGGACGAG	XM_026209143.1
*MyoD*	GTCCAACAACCCCAACCAG	GCTTTTAGTATTCCGTGCGTC	XM_026239323.1
*MyoG*	GACCAATCCCTACTTCTTCGC	TTTGTCCTCCAACCCCACT	XM_026275805.1
*MRF4*	GTCATGTCTAATGTGGGCTTGT	GGATTGGGCACCGTCTTT	XM_026231638.1
*MYF5*	TCTGTAGATACGGGAGTTGCG	ACTGGTCTGCTGCTGGGAC	XM_026209828.1
*LPL*	GGGCTACGGAGTCAACAAAA	CAGGCAAAATGAAGGGGATA	XM_026198434.1
*ADSL*	GGGCTGGTGGTGTATCCTAA	GGCAGTCCTGTCTGTTTCCT	XM_026209452.1
*MyHC*	GTGCTTGACATTGCTGGGTT	ATGCCTTCTTTCTTGTATTCCT	XM_026260328
*ACSL1*	CCCAAAGCACTTAAACCACC	AGATCCCAAACAAGGACCATT	XM_026203856.1
*PPARα*	CCAATACTGCCGTTTCCG	GGGACTCGTGCTCATCTTTTAC	XM_026209687.1
*β-actin*	AAACGACCAACCCAAACC	GACGCTTCTGGAACGACTAA	XM_026258408.1

**Table 4:** Effects of dietary levels of Met and Lys supplementation on growth performance and somatic indexes of Hefang crucian carp.

Group	LA	MA	HA	*p*-Value
IBW (g)	100.37 ± 0.11	100.33 ± 0.09	100.27 ± 0.17	0.875
FBW (g)	154.54 ± 9.58	163.61 ± 14.19	187.45 ± 12.24	0.222
WGR (%)	53.96 ± 9.42	63.08 ± 14.17	86.91 ± 11.88	0.214
SGR (% day^−1^)	0.71 ± 0.1	0.8 ± 0.14	1.03 ± 0.1	0.211
SR (%)	100 ± 0	100 ± 0	100 ± 0	-
CF (%)	2.8 ± 0.06	3 ± 0.04	2.85 ± 0.1	0.175
HSI (%)	2.27 ± 0.21	1.9 ± 0.31	2.78 ± 0.33	0.149
VSI (%)	5.97 ± 0.58	5.76 ± 0.37	7.29 ± 0.36	0.092

Data were analyzed using one-way ANOVA and were expressed as mean ± SEM. The specific *p*-values from the ANOVA are provided in the table. Growth performance indicators (IBW, FBW, WGR, SGR) were calculated based on the average of all fish per tank (*n* = 3 replicates per group), no mortality occurred during the 8-week trial (Survival rate = 100%), and all surviving fish were measured for the analyses. Somatic indexes (CF, HSI, VSI) were measured using 6 individuals randomly sampled from each treatment group (*n* = 6). The abbreviations and formulas are defined as follows: IBW (Initial weight); FBW (Final weight). Weight gain rate (WGR, %) = 100 × (Final fish weight − Initial fish weight)/Initial fish weight. Specific growth rate (SGR) = (Final fish weight − Initial fish weight)/day. Survival rate (SR, %) = 100 × (Final fish number/Initial fish number). Condition factor (CF) = Body weight/(Body length)^3^. Hepatosomatic index (HSI, %) = 100 × (Liver weight/whole body weight). Viscerosomatic index (VSI, %) = 100 × (Viscera weight/whole body weight).

**Table 5:** Effects of dietary levels of Met and Lys supplementation on serum indexes of Hefang crucian carp.

Group	TP (mg/mL)	GLU (mmol/L)	T-CHO (mmol/L)	TG (mmol/L)
LA	22.72 ± 0.52 ^b^	4.01 ± 0.09 ^b^	7.78 ± 0.26 ^a^	4.18 ± 0.1 ^a^
MA	27.78 ± 0.95 ^a^	5.15 ± 0.08 ^b^	5.19 ± 0.18 ^b^	3.56 ± 0.16 ^b^
HA	24.87 ± 1.31 ^b^	10.06 ± 0.91 ^a^	5.06 ± 0.54 ^b^	3.48 ± 0.23 ^b^
*p*-value	0.016	<0.001	<0.001	0.027

TP: total protein, GLU: glucose, T-CHO: cholesterol, TG: triglyceride. The specific *p*-values from the ANOVA are provided in the table. The results are shown as mean ± SEM (n = 6). Different superscript letters in the same column indicate significant differences (*p* < 0.05).

**Table 6:** Comparison of texture measurements of Hefang crucian carp fed different diets for 8 weeks.

Group	Hardness (N)	Cohesiveness (Ratio)	Springiness (mm)	Gumminess (N)	Chewiness (mJ)	Adhesiveness (N.mm)
LA	44.4 ± 2.61 ^b^	0.24 ± 0.02	1.58 ± 0.09 ^a^	10.31 ± 0.81 ^b^	15.83 ± 1.75 ^b^	0.08 ± 0.01
MA	78.18 ± 7.49 ^a^	0.26 ± 0.01	0.93 ± 0.05 ^b^	19.1 ± 0.94 ^a^	15.23 ± 1.96 ^b^	0.05 ± 0.003
HA	58.78 ± 6.65 ^b^	0.25 ± 0.03	1.48 ± 0.12 ^a^	17.2 ± 3.16 ^ab^	27.3 ± 4.79 ^a^	0.07 ± 0.01
*p*-value	0.002	0.715	0.016	0.043	0.035	0.134

The specific *p*-values from the ANOVA are provided in the table. The results are shown as mean ± SEM (*n* = 6). Different letters in the same column indicate significant differences (*p* < 0.05).

## Data Availability

All data generated or analyzed during this study are included in the manuscript and supplementary files. The raw transcriptome sequencing data reported in this paper have been deposited in the NCBI Gene Expression Omnibus (GEO) database under the accession number GSE331020.

## Supplementary Materials

The following supporting information can be downloaded at: https://www.mdpi.com/article/10.3390/ani16111636/s1, Table S1. Summary of RNA-seq data quality metrics for each sample. Table S2. Summary of RNA-seq mapping statistics for each sample.
