# Supplementary material for: Dietary Supplementation with Methionine and Lysine Enhances Antioxidant Function and Muscle Quality of Hefang Crucian Carp (Carassius auratus)
Source: Animals (Basel). 2026 May 27;16(11):1636. doi: 10.3390/ani16111636 (PMC13255624; doi:10.3390/ani16111636)
Supplement: Supplementary file 1 [file animals-16-01636-s001.zip › animals-4285534-supplementary.pdf]

Supplementary information

Table S1 Summary of RNA-seq data quality metrics for each sample.

Sample	Reads No.	Bases (bp)	Q30 (bp)	N (%)	GC (%)	Q20 (%)	Q30 (%)
LA1	49317956	7447011356	7232613350	0.007931	48.16	98.98	97.12
LA2	46735832	7057110632	6868738242	0.007738	48.42	99.07	97.33
LA3	44282136	6686602536	6493464359	0.007835	48.33	98.98	97.11
MA1	49465116	7469232516	7272396690	0.007763	48.50	99.08	97.36
MA2	44849038	6772204738	6571605391	0.007904	48.05	98.95	97.04
MA3	48621566	7341856466	7135434296	0.007851	48.38	99.00	97.19
HA1	43884908	6626621108	6445609373	0.008014	48.17	99.05	97.27
HA2	48836060	7374245060	7160152662	0.007840	48.19	98.98	97.10
HA3	45898244	6930634844	6737499676	0.007622	48.21	99.02	97.21

Note: Sample: sample ID;

Reads No: total number of reads;

Bases (bp): total number of bases;

Q30 (bp), number of bases with a Phred quality score ≥30 (error rate ≤0.1%);

N (%), percentage of ambiguous bases;

Q20 (%): percentage of bases with a Phred quality score ≥20 (error rate ≤1%);

Q30 (%): percentage of bases with a Phred quality score ≥30 (error rate ≤0.1%).

Supplementary information

Table S2. Summary of RNA-seq mapping statistics for each sample

Sample	Clean_Reads	Total_Mapped	Multiple_Mapped	Uniquely_Mapped
LA1	48654158	43106504 (88.60%)	6904331 (16.02%)	36202173 (83.98%)
LA2	46154734	43869117 (95.05%)	9010803 (20.54%)	34858314 (79.46%)
LA3	43672428	40312680 (92.31%)	7510673 (18.63%)	32802007 (81.37%)
MA1	48852128	46493084 (95.17%)	8366302 (17.99%)	38126782 (82.01%)
MA2	44217336	39225319 (88.71%)	6969863 (17.77%)	32255456 (82.23%)
MA3	47969126	43977232 (91.68%)	8261158 (18.79%)	35716074 (81.21%)
HA1	43337312	40924484 (94.43%)	8302612 (20.29%)	32621872 (79.71%)
HA2	48168916	42718760 (88.69%)	6715680 (15.72%)	36003080 (84.28%)
HA3	45312432	41486860 (91.56%)	7729591 (18.63%)	33757269 (81.37%)

Note: Sample: sample ID;

Clean Reads: total number of reads used for mapping;

Total Mapped: total number of reads mapped to the reference genome (percentage calculated as Total Mapped / Clean Reads);

Multiple Mapped: number of reads mapped to multiple locations (percentage calculated as Multiple Mapped / Total Mapped);

Uniquely Mapped: number of reads mapped to a single location (percentage calculated as Uniquely Mapped / Total Mapped).
